# On the Prediction and Optimisation of Processing Parameters in Directed Energy Deposition of SS316L via Finite Element Simulation and Machine Learning

**DOI:** 10.3390/ma18051039

**Published:** 2025-02-26

**Authors:** Mehran Ghasempour-Mouziraji, Daniel Afonso, Ricardo Alves de Sousa

**Affiliations:** 1 TEMA—Centre for Mechanical Technology and Automation, Department of Mechanical Engineering, University of Aveiro, 3810-193 Aveiro, Portugal; dan@ua.pt (D.A.);; 2 LASI—Intelligent Systems Associate Laboratory, 4800-058 Guimarães, Portugal

**Keywords:** directed energy deposition, SS316L, finite element simulation, machine learning

## Abstract

In the current study, the integration of finite element simulation and machine learning is used to find the optimal combination of processing parameters in the directed energy deposition of SS316L. To achieve this, the FE simulation was validated against previously implemented research, and a series of simulations were conducted. Three inputs, namely laser power, scanning speed, and laser beam radius, and two outputs, namely residual stress and displacement, were considered. To run the machine learning model, artificial neural networks and a non-dominated sorting genetic algorithm were applied to determine the optimal combination of processing parameters. In addition, the current study underscores the novelty of combining FE simulation and machine learning methods, which provides enhanced precision and efficiency in controlling residual stress and displacement (geometrical deviation) in the Directed Energy Deposition (DED) process. Then, the results obtained via machine learning were validated with confirmatory tests and reported. The findings offer a practical solution for process parameter optimization, contributing to the progression of additive manufacturing technologies.

## 1. Introduction

Directed Energy Deposition (DED) is an Additive Manufacturing (AM) method that involves melting materials and depositing them layer by layer to produce, repair, or enhance the mechanical and microstructural properties of parts [1]. In this advanced manufacturing process, the molten material is fed in the form of powder or wire onto the substrate to create a melt pool by using different energy sources such as laser, electron beam or plasma arc. This method is highly beneficial, as it reduces material waste and can work with a wide range of materials, including metals, composites and ceramics. DED is widely used in various applications including the aerospace [2], medical [3], automotive [4], and tooling industries [5], fabricating functionally graded structures [5], complex [6], and customized parts, as well as its suitability for repairing damaged components [7].

Despite these advantages, such as high deposition rate, utilizing a wide range of materials, and design flexibility, DED presents some challenges that restrict the fabrication process and efficiency. The first drawback of this method is the poor surface quality [8] of deposited parts which mostly requires post-processing such as machining and grinding. This extra step enhances the time and cost associated with DED. Another challenge in this process is thermal gradient [9], which creates residual stress that can lead to warping and if not properly controlled, affecting the dimensional accuracy and mechanical performance of the fabricated part [10].

In addition, cracking, and other structural defects may occur due to the accumulation of residual stress [11]. These defects can compromise the mechanical integrity of the final product. Although this optimized fabrication method offers various advantages, DED process optimization is a challenging issue that is affected by processing parameters such as laser power, scanning speed, laser beam, and layer thickness. These parameters affect the mechanical and microstructural properties and surface finish. The traditional trial-and-error method for optimization is a time-consuming process and highlights the need for more efficient and systematic approaches. Among various optimization approaches, Machine Learning (ML) has recently been accentuated by industries and academics. Machine learning offers advanced solutions to these challenges by enabling data-driven prediction and optimization of processing parameters [12]. ML algorithms analyze large datasets to find patterns and relationships that are not readily apparent through conventional analytical methods. Regarding DED, ML can be employed to model the complex interactions between processing parameters and resultant material properties, thereby facilitating the prediction and control of the deposition process. Much research has been carried out in the field of optimization of manufacturing processes, particularly the DED process. M. Moradi et al. [13] investigated the Direct Laser Metal Deposition (DLMD) method for the additive manufacturing of Inconel 718 Ni-based superalloy using a full factorial design approach. They focused on how three process parameters, namely laser scanning speed, powder feed rate and scanning strategy, affect the geometrical dimensions, microhardness, and stability of the manufactured walls. They found that lower scanning speeds and higher powder feed rates resulted in increased wall height and width. The microhardness elucidated variability due to microstructural phases. The optimal combination was found to be at low scanning speeds with a unidirectional scanning pattern, accounting for enhanced melting time and smoother surfaces. P. Pant et al. [14] investigated the Laser Metal Deposition (LMD) process for surface coating utilizing SS316L material. Artificial Neural Networks (ANNs) and a combined Particle Swarm Optimization (PSO)-ANN model were used to predict the main outputs such as clad layer height, width, and powder capture efficiency. Before running optimization via (PSO)-ANN, Response Surface Methodology (RSM) was used to gather data. These findings demonstrated that the PSO-ANN model offers superior reliability and accuracy for predicting and optimizing LMD process parameters compared to traditional empirical methods and simple ANN models. M. Pellizzari et al. [15] optimized the process of depositing single-layer H13 tool steel cladding on a copper-beryllium (CuBe) alloy substrate using direct laser metal deposition (DLMD), by considering laser power, powder feed rate, overlap ratio, and substrate temperature. The quality of cladding was evaluated by defects, interfacial adhesion, geometrical features and substrate over-ageing, and it is worth noting that it was affected by CuBe’s high reflectivity. Optimal combination of parameters fabricated dense cladding, with substrate preheating preventing cracks but causing softening at higher temperatures: 13% hardness reduction at 150 °C and 40% at 250 °C. R. Mahamood et al. [16] explored the effect of processing parameters on material usage efficiency in LMD of Ti6Al4V using a two-level full factorial design of experiments. Laser power, scanning speed, powder flow rate and gas flow rate are the processing parameters. The optimal combination of processing parameters is as follows: laser power of 3.2 kW, a scanning speed of 0.06 m/s, a powder flow rate of 2 g/min, and a gas flow rate of 3 L/min.

R. Li et al. [17] examined the deformation distribution and residual stress in the fabrication of thin-walled Al-Cu alloy components using a dual-robot collaborative wire-arc additive manufacturing (WAAM) ((FANUC M-20iA, Echternach, Luxembourg) paired with a welding unit (Fronius CMT Advanced 4000R, Wels, Austria)). To validate FE simulation, infrared thermography and structured light sensors were used. Both systems showed symmetric stress distributions, with stress concentrations achieved at the junction between the substrate and the thin wall. In addition, the dual-robot WAAM system demonstrates superior performance over the single-robot system, offering reduced temperature gradients, more uniform stress distributions, and less deformation.

**Figure 1:**
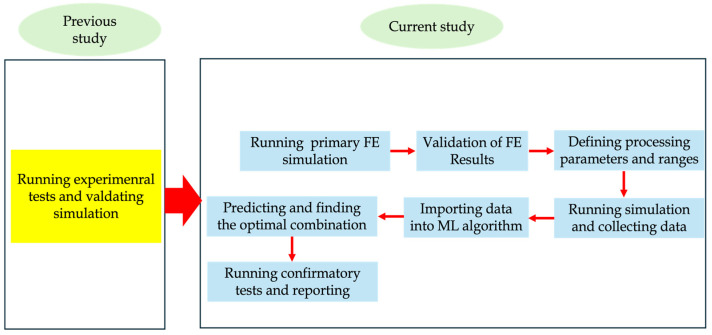
Flow chart of the current study.

Chi Wu et al. [18] used an ML-based design for biomedical engineering ceramic with additive manufacturing to optimize functionally graded tissue scaffolds with Triply Periodic Minimal Surfaces (TPMSs) for bone regeneration. By combining Bayesian optimization and Lithography-based Ceramic Manufacturing (LCM), the framework achieves improved bone ingrowth and precise geometric qualities, offering a novel tool for practical design optimization in ceramic AM. H. Mu et al. [19] investigated an adaptive online simulation model for Wire Arc Additive Manufacturing (WAAM), addressing challenges in distortion prediction for building a Digital Twin (DT). Utilizing Vector Quantized Variational AutoEncoder coupled with Generative Adversarial Network (VQVAE-GAN) architecture for spatial feature extraction and an RNN for time-scale fusion, the model estimates distortion fields online with high accuracy, significantly outperforming traditional FEM and ANN methods. T. Loreau et al. [20] addressed the complexity of simulating porosity in Wire Laser Metal Deposition (WLMD) due to the intricate interactions between the laser and material and the varying scales of time and space involved, which significantly affect simulation efficiency and performance. To tackle this challenge, they proposed utilizing neural networks to model porosity as a function of processing parameters. Firstly, the relevant parameters were found and analyzed for their effect on porosity. Once the correlations were confirmed, these parameters were used as inputs for constructing and training a Multilayer Perceptron (MLP) neural network with part of the available data. The model’s performance was then evaluated using both the training dataset and a separate test dataset to assess its predictive capabilities. In the current study, the prediction and optimization of DED processing parameters are achieved using an ML approach and finite element simulation. The primary objective of this study is to develop robust predictive models for the key output variables of residual stress and geometrical deflection (nodal displacement) and to optimize the process parameters to achieve the desired performance outcomes. Figure 1 shows the flow chart of the current study.

## 2. Finite Element Simulation and Validation

**Figure 2:**
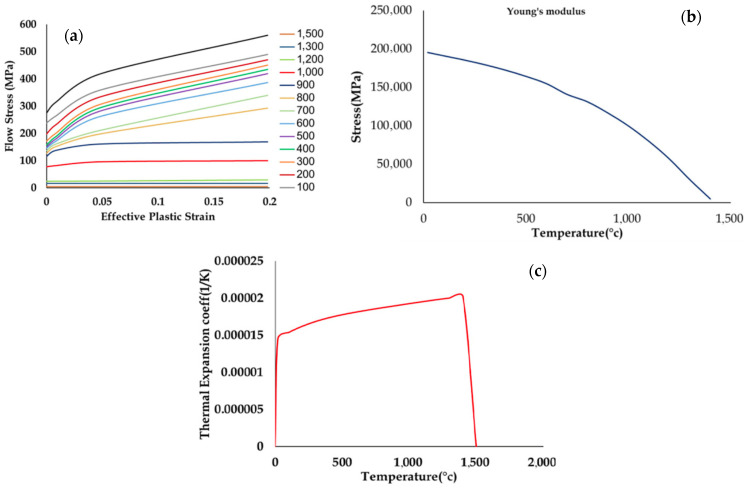
(**a**) Temperature-dependent material properties of SS316L adapted from Simufact Welding (Flow stress) [25], (**b**) Temperature-dependent material properties of SS316L adapted from Simufact Welding (Young’s Modulus), (**c**) Temperature-dependent material properties of SS316L adapted from Simufact Welding (thermal expansion) [25].

To simulate the DED process, MSC APEX 2023 and Simufact software 2023 were used. Firstly, the parts were designed in MSC Apex and then imported into the Simufact Welding Module, which applies temperature-dependent material properties that alter the microstructural characteristics with each heating cycle. The cubic element size for the substrate is 2.91 × 3 × 2.91 mm^3^ with a total substrate size of 100 × 100 × 6 mm^3^ and the element size for layers is 0.6 × 1.2 × 0.715 mm. The current study defines a range for the processing parameters, ensuring that the chosen values are representative operating conditions. By using this range, we aim to explore and identify optimal combinations of processing parameters. During the simulation, thermo-mechanical behavior was modeled throughout the transient deposition process using a quasi-static element formulation. The melt pool and Marangoni effect were modeled using a low-stiffness solid approach and artificial thermal conductivity, respectively. Table 1 and Table 2 show the chemical and mechanical properties of SS316 [21,22,23]. Also, Figure 2 shows the Temperature-dependent material properties of SS316L. The part was built by deposition of 20 layers with different processing parameters, which are given in Table 3. The process parameters included in Table 3 were selected based on a previous study used to validate the simulation

**Table 1:** Chemical composition of ss316 [24].

Grade	C	Mn	Si	P	S	Cr	Mo	Ni	N
min	-	-	-	-	-	16.0	2.00	10.0	-
max	0.03	2.0	0.75	0.045	0.03	18.0	3.00	14.0	0.10

**Table 2:** Mechanical properties of SS316 [10].

Parameter	Value
Solidus temperature	1279 °C
Melting temperature	1450 °C
Density	7966 Kg/m^3^
Latent heat for melting	25,400 J/Kg
Poison ratio	0.3
Convective heat transfer for first and remaining layers	10, 30 (W/m^2^K)
Emissivity for first and remaining layers	0.6, 0.6
Elastic module	200 GPa
Ultimate Tensile Strength	580 MPa
Yield Strength	290 MPa
Specific heat	2051.531 J/kg. °C
Thermal conduction	0.225 W/cm. °C

**Table 3:** Process parameters.

Parameter	Value
Laser power	400–800 W
Scanning speed	10–20 mm/s
Laser beam diameter	0.6–0.8 mm
Powder feed rate	7.5 g/min
Powder size	45–90 µm

**Figure 3:**
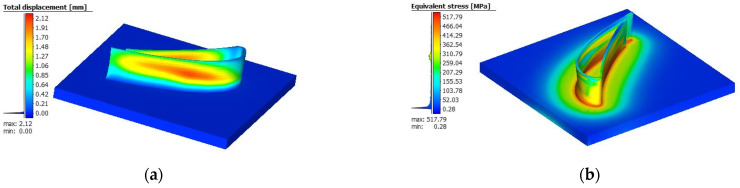
FE simulation of current study (**a**) displacement and (**b**) residual stress.

To validate the simulation, a 50 mm bead was deposited using the same process conditions as previous research [26]. Since in the current study, residual stress and maximum nodal displacement on the sample (deflection) were used, the FE simulation results for those outputs are given in Figure 3.

## 3. Machine Learning Procedure

**Figure 4:**
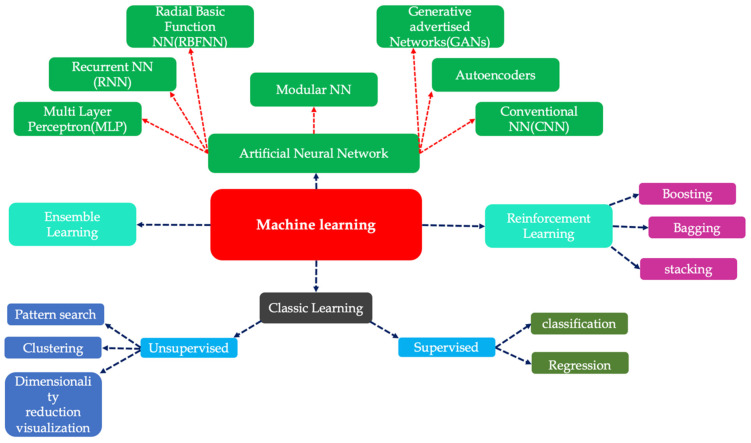
Methods of machine learning.

Machine Learning (ML) is a subset of Artificial Intelligence (AI) that focuses on developing algorithms and statistical models enabling computers to learn from and make decisions based on data [27]. It has been accentuated as a powerful method in many fields, such as banking [27], healthcare [28], image processing and identification [29,30] and engineering [31,32]. Traditional programming requires developing the rules and instructions to handle the tasks specifically, but ML has no need for any explicit programming, and computers recognize the patterns, make choices, and predict and optimize the data automatically. ML consists of various methods and techniques for training algorithms to learn from data and make predictions or decisions autonomously, as shown in Figure 4.

## 4. Multi-Objective Optimization of Processing via ML

Multi-objective optimization has enormous practical importance. Almost all optimization challenges that occur in realistic situations may be characterized as having several different objectives. Usually, there is no single optimal solution in multi-objective optimization, also known as vector optimization, that simultaneously fulfills all objective functions. Thus, a set of solutions known as Pareto optimal solutions emerges. Pareto optimal solutions represent the best possible values for the objectives without favoring one objective over another.

In Pareto-based multi-objective optimization, the population is arranged onto the Pareto front, and the individuals are rated according to how superior they are. This process can be defined as [33]:

(1) $$F\;(X)={\left[f_{1}(X),\;\;f_{2}(X),\ldots ,\;\;f_{k}(X)\right]}^{T}$$

represents the vector of objective functions, and $X^{*}\in {ℜ}^{n}$ is the vector of design variables. Subject to m inequality constraints [34]:

(2) $$L_{i}\;(X)\leq 0,\;\;\;\;i=1\;\;to\;\;m$$

and p equality constraints [34]:

(3) $$H_{j}\;(X)=0,\;\;\;j=1\;\;to\;\;p$$

When Solution A obtains a lower value (in the case of minimization) for at least one target and does not perform worse than Solution B for the remaining objectives, it outperforms Solution B. On the other hand, a point (Ω is a feasible region in ${ℜ}^{n}$ satisfying Equations (2) and (3)) dominated all X∈Ω if and only if [33]:

(4) $$\forall i\in \{1,\;\;2,\ldots ,\;\;k\},\;\;\;\;\forall X\in \Omega -\{X^{*}\}\;\;\;\;\;f_{i}\;(X^{*})\leq f_{i}\;(X)\;\;\wedge \;\;\exists \;j\in \{1,\;\;2,\ldots ,\;\;k\}:\;\;\;\;f_{j}\;(X^{*})<f_{j}\;(X).$$

**Figure 5:**
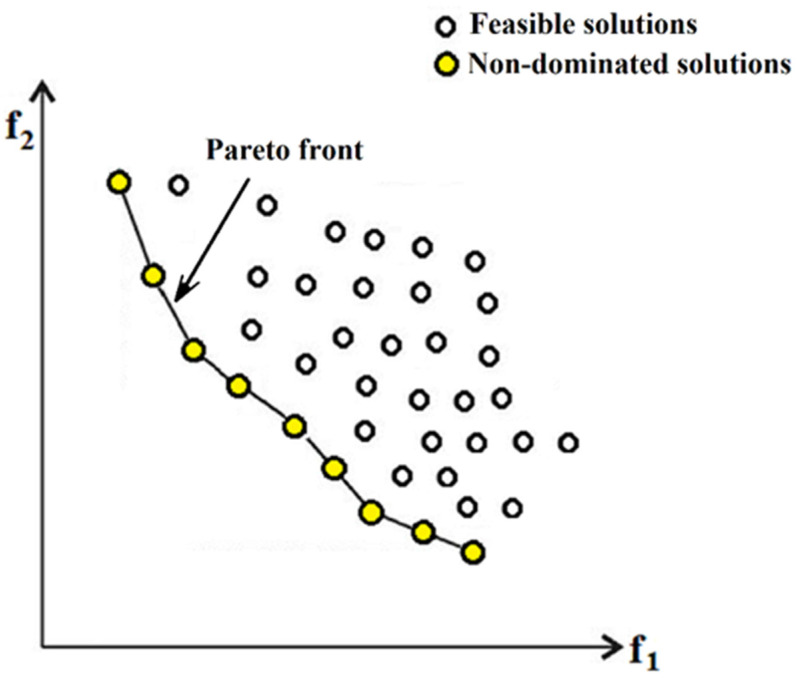
Pareto front in multi-objective optimization algorithms.

According to Figure 5, non-dominated solutions are the ones that are much closer to the optimal solution compared to feasible solutions. Moreover, individuals laid on the “Pareto front” have no superiority over each other.

## 5. Artificial Neural Network (ANN)

**Figure 6:**
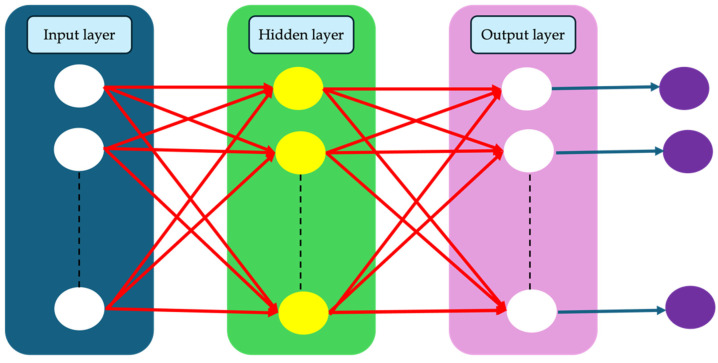
A schematic view of a one-hidden-layer artificial neural network (ANN).

ANN is commonly used as a model to address engineering challenges. Neural networks are composed of an array of neurons, which serve as simple processing units. Typically, an ANN consists of input, output, and hidden layers, with the number of hidden layers varying based on the process and structure of the network. In this setup, neurons are interconnected to neurons in the next layers via direct connection links with assigned weights. It is worth noting that the weight values are adjusted to learn and find the optimal solution for each problem [35]. The number of neurons in both the input and output layers corresponds to the number of physical variables and targets. However, in the hidden layer, the number of neurons may vary based on the complexity of the problem. Determining the optimal network requires assigning an appropriate number of neurons to each hidden layer. In the literature, a feedforward neural network is a commonly applied model, with the Back Propagation (BP) network being widely used in feedforward architectures. A schematic view of ANN is shown in Figure 6.

## 6. Data-Driven Multi-Objective Optimization of Artificial Neural Network

One of the most challenging issues is finding the best ANN with the lowest training and testing errors. In this case, Yarmohammadi et al. [36,37] utilized multi-objective optimization to determine the most optimal ANN with minimal training and testing errors. The primary goal of this section is to construct a multilayer feedforward neural network using the Back Propagation (BP) learning algorithm to predict optimal input parameters while optimizing two conflicting objectives: training error and testing error. The achievement is shown in Table 4 and Table 5. In the input layer of ANNs, there are three input parameters, namely laser power, laser beam radius, and scanning speed, of which the range is given in Table 3. In the current study, the input parameters of ANN and their variables are presented in Table 2. The output parameters include residual stress and displacement, which are considered the first target and second target, respectively. The layers between the input and output layers are known as hidden layers. Initially, several attempts were made to find an optimal ANN configuration that minimized all targets simultaneously; however, this approach resulted in high test and training errors. To address this issue, a unique ANN was developed for each output, which helped reduce both training and testing errors.

In this study, a two-layer network with a maximum number of neurons of 30 per layer is proposed as it has the capability to find the required optimum ANN configuration. Raising the number of layers as well as neurons could result in a negligible improvement in the result, but could also lead to significant increases in the computation cost. There were two optimum configurations for the first target and only one configuration for the second target. In the case of the first target, comparing the output of these two configurations did not show any meaningful difference. Thus, an output of only one of them was reported in this research.

The given information in Table 4 is crucial for training the ANN model and affects its performance in terms of both training and test error. The back propagation algorithm is used to train the ANN, which consists of three approaches TrainLM (Levenberg–Marquardt), TrainBR (Bayesian Regularization) and TrainCGF (Conjugate Gradient with Fletcher–Reeves). TrainLM (Levenberg–Marquardt) is a fast, widely used algorithm for training networks, typically providing high accuracy with fewer iterations. The number of Neurons in 1st Layer refers to the number of neurons in the first hidden layer of the network. Each neuron in this layer processes a different feature of the input data. The number of Neurons in 2nd Layer specifies the neurons in the second hidden layer, where the network starts to learn more complex features from the data. Transfer Function in 1st Hidden Layer defines how the output of each neuron in the hidden layer is calculated. In the current study, the **Tan-sig** (tangent sigmoid) transfer function is utilized in the first layer, which outputs values between −1 and 1, helping the network to learn complex patterns. Transfer Function in 2nd Hidden Layer used in the second hidden layer is **Log-sig** (logistic sigmoid), which outputs values between 0 and 1. This function is particularly useful for binary classification tasks or probabilities. Train Error (%) represents the error (in percentage) during the training phase, showing how well the network fits the training data. The lower the training error is the better in learning patterns. Test Error (%) shows the error (in percentage) when the network is examined based on the data that have not been used in the training section. This aids in assessing how well the model generalizes to new, unseen data.

**Figure 7:**
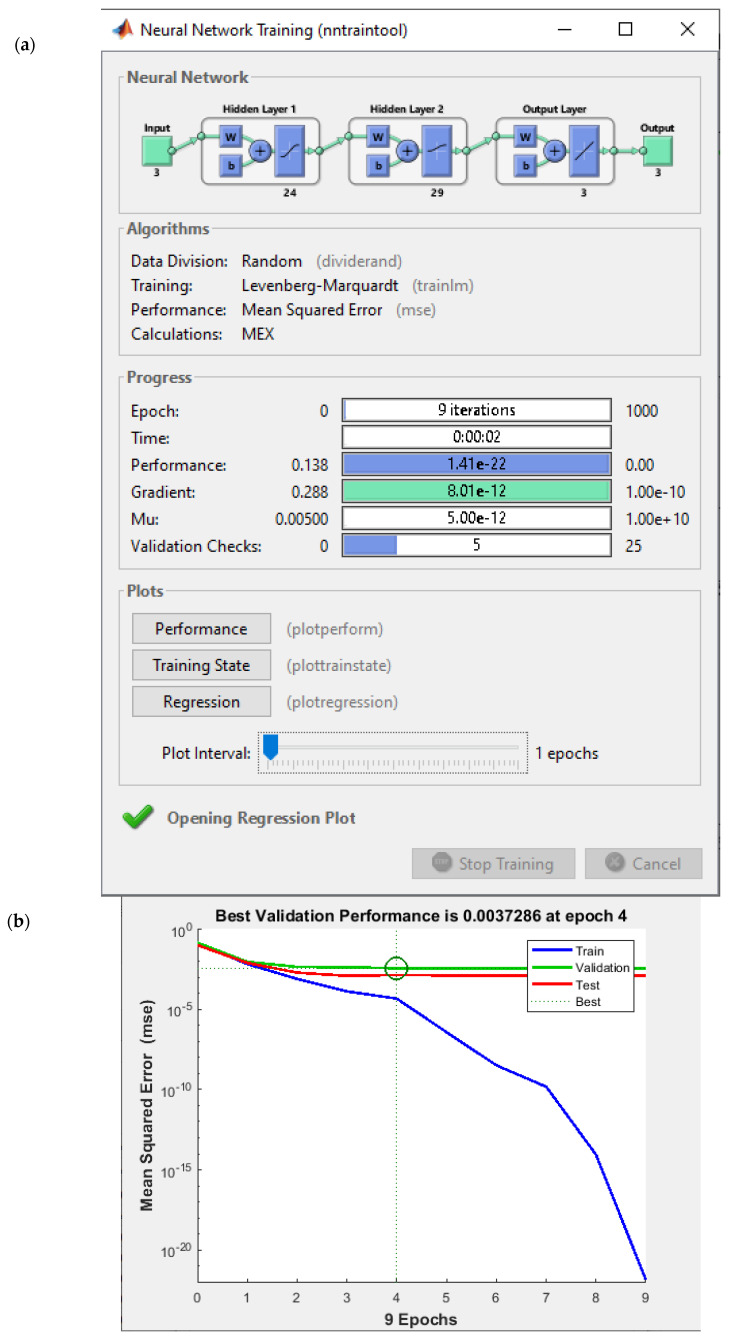

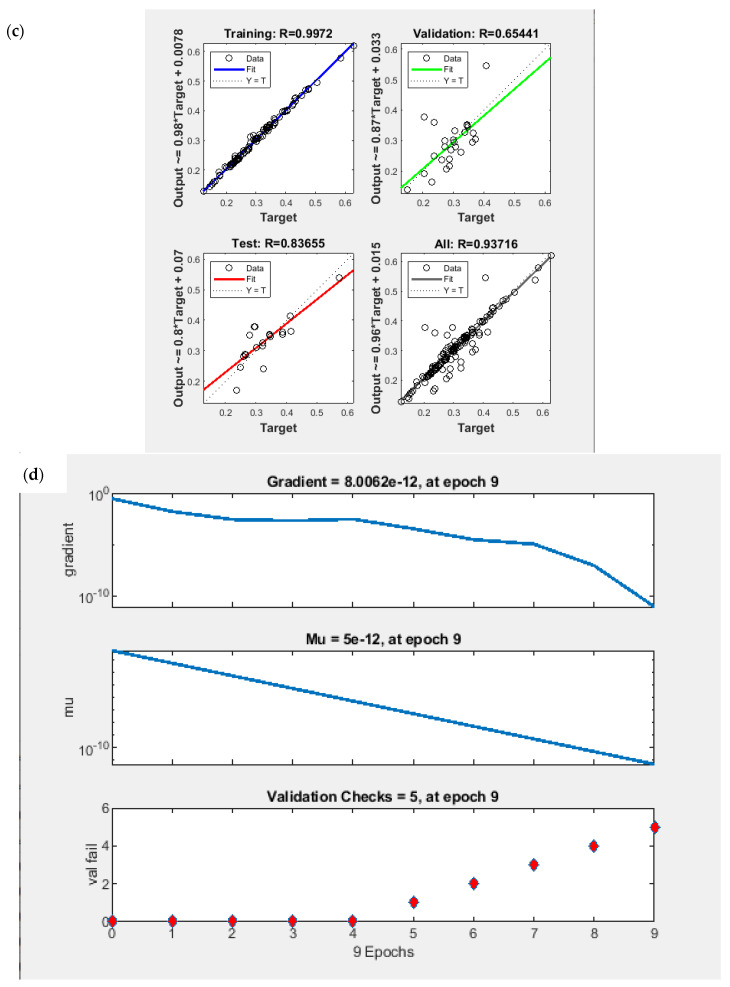
(**a**) Performance of ANN, (**b**), best validation performance, (**c**) Train, validation, and test regression of ANN, (**d**), training state performance plot (for first target).

The performance and train, validation, and test regression graph for the first target is illustrated in Figure 7.

**Figure 8:**
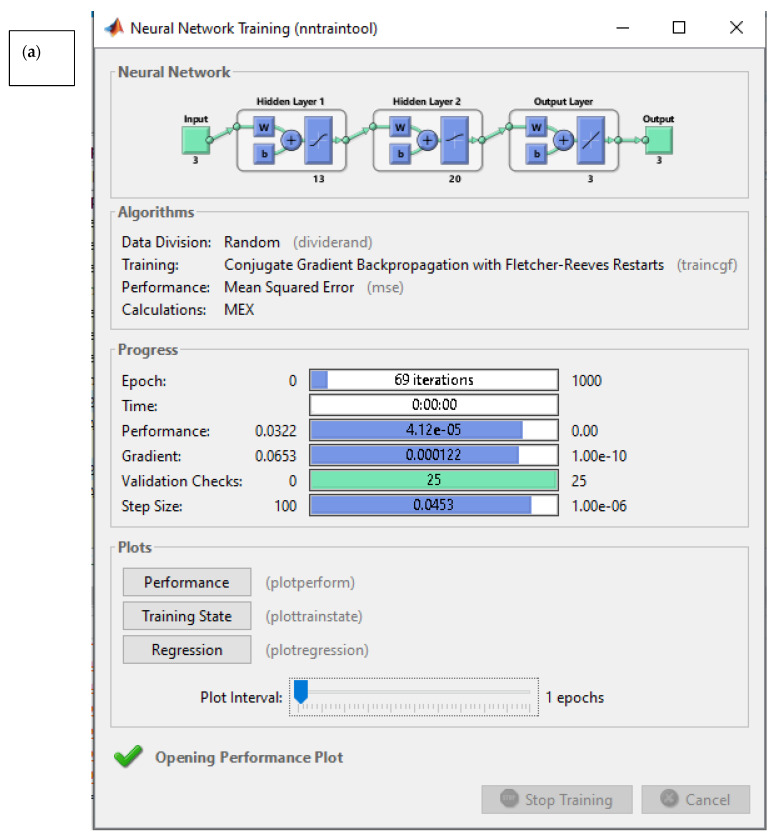

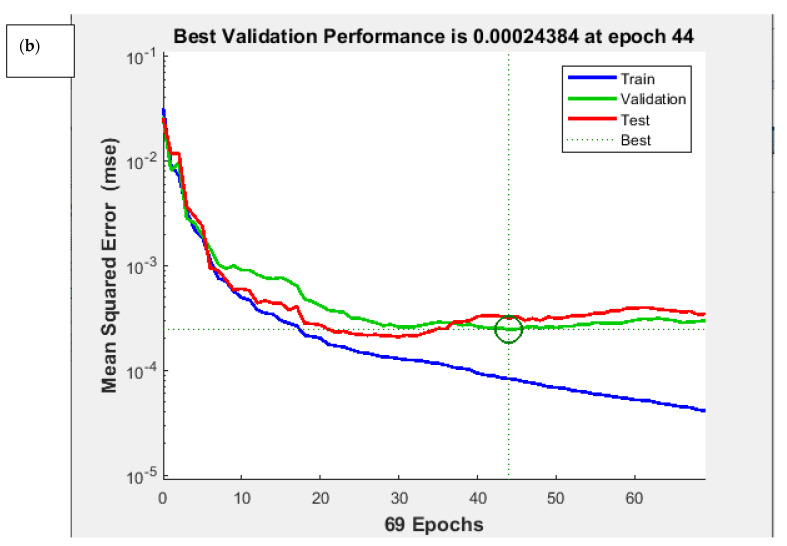

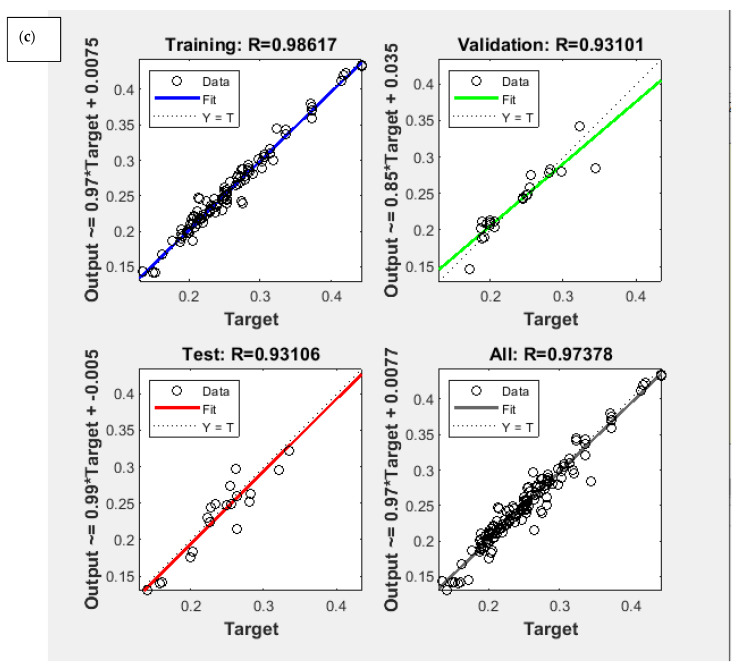

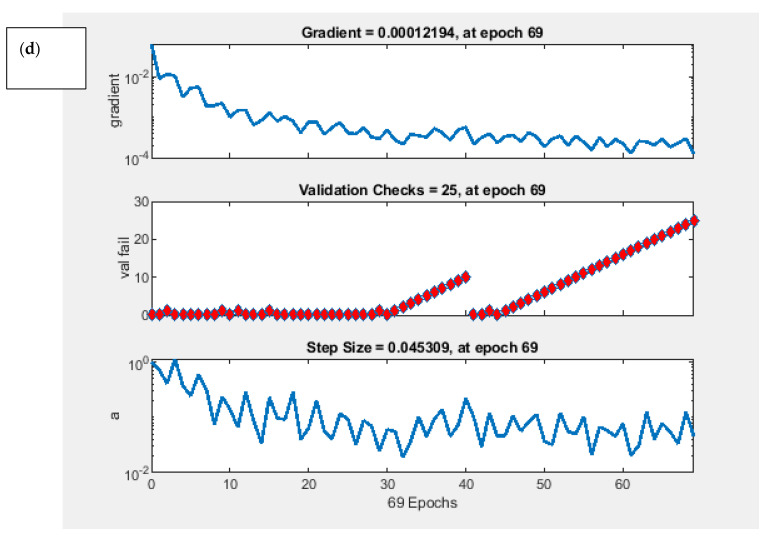
(**a**) Performance of ANN, (**b**), best validation performance, (**c**) train, validation, and test regression of ANN, (**d**), training state performance plot (for second target).

Also, the information for ANN for the second target is shown in Table 5 and related information about the performance of ANN is shown in Table 5. Also, the performance of second target is shown in Figure 8.

The first stage of optimization is generating a random population through iteration toward the best solution. The detailed methodology to determine the optimal ANN is given in [36,37], where the authors give detailed information about the involved steps. The related processing parameters are shown in Table 6.

To find the final objective of each individual chromosome, 10 independent runs were carried out. This approach helps to eliminate the unstable chromosome from consideration. The evolutionary process of selecting the optimal network variable vectors to identify the Pareto front of two conflicting objectives (training and testing errors) is conducted using the NSGA approach. The optimal artificial neural networks were identified for each output individually, and their corresponding training and testing errors are presented in Table 4 and Table 5.

## 7. Multi-Objective Optimization of Processing Parameters in DED

**Figure 9:**
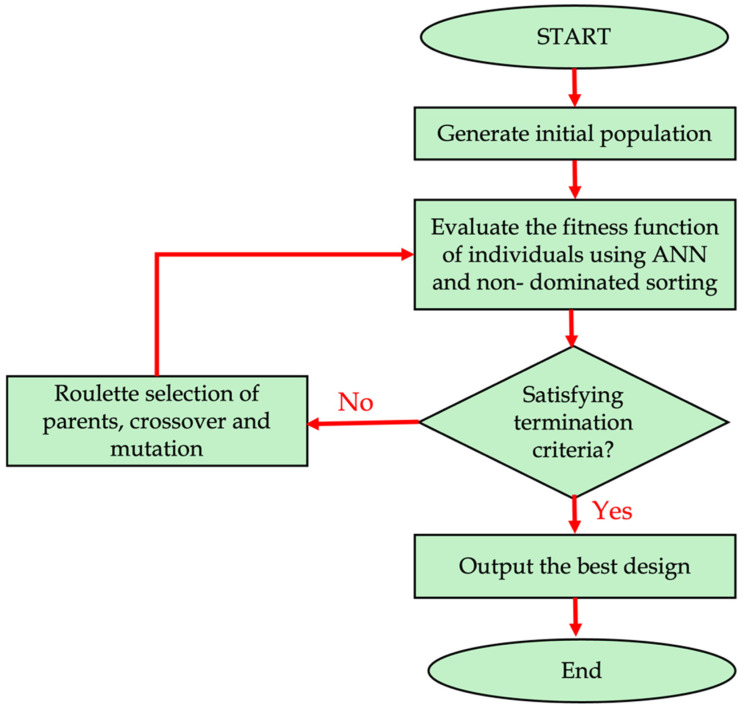
The NSGA-II algorithm.

Having found the optimal ANN for each target, in the next step, the best results (lowest values) are found in the continuous domain bounded. Thus, NSGA was utilized to determine the optimal inputs, resulting in the optimal combination of laser power, scanning speed, and laser beam radius. Figure 9 illustrates the NSGA algorithm utilized in this study. The non-dominated sorting genetic algorithm, NSGA-II, is one of the best evolutionary algorithms for the optimization of multi-objective problems, which has been employed in several studies. Since the sorting method employed in this algorithm introduces all possible scenarios, NSGA guarantees the obtainment of the appropriate combination of parameters leading to the optimum output. The algorithm starts generating a random initial population. Then, using operators such as crossover and mutation, it tries to find the best input parameters until satisfying the termination criteria. The termination criteria could be a certain number of iterations (as used in this study), reaching a certain and acceptable output, or any other criteria a designer determines. Additionally, Table 7 demonstrates the results achieved through the ANN. To select the best option among these individuals, their values referring to the targets were compared and scored 1 to 4 based on their strengths.

Table 7 presents the optimal values for the current research derived from NSGA. These values provide information about the best combination of processing parameters that lead to an optimal output for the DED process. It is worth noting that the achieved results are in the predefined range of input processing parameters and corresponding outputs according to Table 3. This ensures that the optimization process stays within feasible and practical limits.

Regarding the effect of processing parameters, increasing laser power enhances the melt pool size which increases the higher material deposition volume in the deposition area [38]. This leads to overbuilding, which causes geometrical deviation. In addition, higher laser power causes deeper penetration on the substrate or previous layers causing irregularities on the surface [39]. Furthermore, keyholing may occur, leading to defects such as pores and voids. The effect of laser power on residual stress can be attributed to the high thermal gradient between the melt pool and surrounding materials, which increases residual stress [40]. Higher cooling rates and rapid solidification associated with high laser power further contribute to the formation of residual stress.

By enhancing scanning speed, the delivered energy to the substrate per unit decreases, which creates a smaller thermal gradient followed by lower residual stress due to a small difference between the melt pool and surrounding materials [41,42]. In addition, while less material is deposited to the melt pool, incomplete fusion happens between the layers, causing defects such as porosity or gaps leading to geometrical deviation. Also, a higher scanning speed can reduce heat accumulation to control distortion.

By increasing the laser beam radius, the residual stress decreases, because the larger laser beam spreads the energy on the wider area which leads to lower energy density [43]. With lower energy density, the thermal gradient is reduced, followed by more uniform heating and cooling. On the other hand, increasing the laser beam radius is likely to create a higher deviation in the deposited area. The larger beam size increases the size of the melt pool which decreases the precision and increases geometrical deviation.

## 8. Conclusions

In the current study, the integration of FE simulation and machine learning, particularly Artificial Neural Networks (ANN) and Non-sorting Genetic Algorithms (NSGAs), was used to find the optimal combination of processing parameters in the directed energy deposition of SS316L to evaluate the residual stress and deflection (displacement). The focus was to assess residual stress and displacement during the process.

1. **Key findings:** Firstly, the validation process was successfully implemented and then a series of tests were carried out to create the data for ANN and NSGA to find the optimal combination of laser power, scanning speed and laser beam radius to reduce the residual stress and displacement. The application of ANN and NSGA enabled the identification of an optimal combination of processing parameters
2. **Main Advantages:** The achievement from the current study illustrated significant enhancement in controlling residual stress and geometrical deviation. This highlights the potential of combining FE simulation with ML algorithms to enhance the efficiency and precision of the DED process, offering a more accurate approach to process optimization.
3. **Implications for Future Research:** The obtained results serve as an applicable reference for further process validation and refinement, particularly when compared to experimental data. This research contributes to a deeper understanding of how different processing parameters affect the overall thermo-mechanical behavior of the DED process, providing insight for future advancements in additive manufacturing.

## Figures and Tables

**Table 4:** Variables in multi-objective optimization of artificial neural network for the first target.

	Back Propagation Algorithm	Number of Neurones in 1st Layer	Number of Neurones in 2nd Layer	Transfer Function in 1st Hidden Layer	Transfer Function in 2nd Hidden Layer	Train Error (%)	Test Error (%)
A	TrainLM	24	29	Tan-sig	Log-sig	4.326	7.760
B	TrainBR	20	10	Tan-sig	Log-sig	4.412	7.160
C	TrainCGF	20	9	Tan-sig	Log-sig	4.332	7.566

**Table 5:** Variables in multi-objective optimization of artificial neural network for the second target.

	Back Propagation Algorithm	Number of Neurones in 1st Layer	Number of Neurones in 2nd Layer	Transfer Function in 1st Hidden Layer	Transfer Function in 2nd Hidden Layer	Train Error (%)	Test Error (%)
A	TrainCGF	13	20	Tan-sig	Log-sig	2.518	3.782

**Table 6:** Detailed information about the NSGA.

Parameter	Value
population size	40 chromosomes
mutation rate	0.1
crossover rate	0.85 over 40 generations
Execution time for each network	10 times

**Table 7:** Obtained results through the NSGA and validation.

Laser Power (W)	Scanning Speed (mm/s)	Laser Beam Radius (mm)	Displacement (mm) (NSGA)	Residual Stress (NSGA)	Displacement (mm) (FE Simulation)	Residual Stress (FE Simulation)
684.93	10.46	0.59	1.56	450.57	1.62	489
713.12	10.73	0.57	1.46	457.17	1.51	491

## Data Availability

The original contributions presented in this study are included in the article. Further inquiries can be directed to the corresponding author.

## Nomenclature

Directed Energy Deposition	DED	Non-dominated Sorting Genetic Algorithm	NSGA
Machine Learning	ML	Laser Metal Deposition	LMD
Finite Element	FE	Direct Laser Metal Deposition	DLMD
Artificial Neural Network	ANN	Particle Swarm Optimization	PSO
Multilayer Perceptron	MLP	Wire Laser Metal Deposition	WLMD
Artificial Intelligence	AI	Back Propagation	BP
